# Cognitive challenge as a probe to expose sex- and age-related differences during static contractions

**DOI:** 10.3389/fphys.2023.1166218

**Published:** 2023-05-16

**Authors:** Hugo M. Pereira, Sandra K. Hunter

**Affiliations:** ^1^ Department of Health and Exercise Science, The University of Oklahoma, Norman, OK, United States; ^2^ Department of Physical Therapy, Marquette University, Milwaukee, WI, United States

**Keywords:** fatigue, aging, sex difference, gender difference, force steadiness, cognitive stress, motor unit, dual-task

## Abstract

Despite activities of daily living being frequently performed simultaneously with a cognitive task, motor function is often investigated in isolation, which can hinder the applicability of findings. This brief review presents evidence that 1) performing a cognitive challenge simultaneously with a motor task can negatively impact force steadiness and fatigability of limb muscles during a static contraction, 2) the negative impact on old adults (>65 years old), particularly older women is greater than young when a cognitive challenge is simultaneously performed with a static motor task, 3) age-related mechanisms potentially explain impairments in motor performance in the presence of a cognitive challenge, and 4) the mechanisms for the age-related decrements in motor performance can be distinct between men and women. These observations are highly relevant to the older adults, given the increased risk of accidents and injury when a motor task is performed with a high cognitive-demand task, especially in light of the expanding reliance on an aging workforce.

## Introduction

Motor function differs between the sexes and declines with age (Hunter et al., 2016). Two important attributes of motor function, for example, are holding steady contractions and fatigability of limb muscles. Considerable progress in understanding the sex and age-related differences and their underlying mechanisms has occurred over the last 20 years by studying these attributes independently from cognitive function [for review (Kluger et al., 2013; Schrack et al., 2020; Enoka and Farina, 2021)]. However, many work-related tasks and activities of daily living are rarely executed in isolation (e.g., typing simultaneously when watching a lecture and performing error corrections) and optimal functional performance often requires increased cognitive function during a motor task. Additionally, recent developments in technologies prompting constant human interaction (e.g., driving while simultaneously operating a virtual assistant using a voice interface) bring an extra layer of complexity to motor tasks, particularly if cortical networks are shared between activities (Mac-Auliffe et al., 2021; Walker et al., 2021). Some motor tasks require static (i.e., isometric) sustained and repeated muscle contractions performed during prolonged periods in the presence of heightened levels of arousal and stress depending on the environment.

In the laboratory setting, experimental models have mimicked these stressful conditions found in daily activities or sport events by imposing a cognitive task before or during the motor task of interest (Koch et al., 2018; Pageaux and Lepers, 2018). The cognitive tasks used typically target working memory, processing speed, cognitive flexibility and executive function, which are important constructs required in daily activities. By using these controlled experimental models, it is possible to non-invasively challenge aspects of the neuromuscular system that are frequently required in sports or daily tasks, as well as to investigate the physiological adaptations involved in the presence of the cognitive task. For example, in young individuals it was observed a reduction in shoulder moments when a difficult cognitive task was performed simultaneously with a static shoulder abduction task (MacDonell and Keir, 2005), as well as modulation of proximal muscles activity (e.g., upper trapezius) (Waersted and Westgaard, 1996). These physiological alterations have potential implications for daily activities that depend on static contractions of proximal muscles to support dynamic activity of distal joints.

Motor and cognitive function diminishes with aging with more rapid declines after 65 years (Grady, 2012; Hunter et al., 2016), with important implications for older men and women, who remain in the workforce longer than previously (Goldin and Katz, 2018; Bureau of Labor Statistics, 2021), and particularly for women who have greater longevity than men (Kochanek et al., 2020). Age-related changes that affect motor function are reviewed in detail elsewhere (Aagaard et al., 2010; Hepple and Rice, 2016; Hunter et al., 2016; Cruz-Jentoft et al., 2019) but briefly include adaptations at all levels of the neuromuscular system, such as apoptosis of spinal neurons, changes in properties of existing motor units as well as reductions in the muscle fiber size and number. All these factors contribute to the loss of muscle force, slowing of movements, declines in muscle power, increased variability of motor task performance, and reduced overall physical function. Cognitive function is also altered by age-related adaptations in the brain (Spreng and Turner, 2019). Older adults typically have difficulties in tasks requiring episodic memory and executive function due to a myriad of age-related brain adaptations that include thinning of the frontal lobes as well as losses in the integrity of the corpus callosum and hippocampus (Grady, 2012). Adequate performance of motor activities simultaneously with a cognitive task depend on the integrity of all these structures that are largely affected by aging.

This brief review will highlight the impact of increased cortical input via imposing a challenging cognitive task during fatiguing and non-fatiguing static contractions in young and older men and women. We focus on force steadiness and time to task failure of limb muscles. The evidence summarized in this review suggests that imposing a cognitive challenge during a motor task can expose sex and age differences in motor performance that are subclinical and not typically observed in the absence of cognitive challenge. Exposing these sex and age-related differences in motor function when simultaneously performing cognitive and a motor tasks, and understanding the physiological mechanisms involved are relevant for designing interventions to improve performance during similar work-related tasks.

## Cognitive challenge and performance fatigability

Fatigue is a disabling symptom in which physical and cognitive function are limited by interactions between performance fatigability and perceived fatigability (Enoka and Duchateau, 2016; Schrack et al., 2020). Quantifying performance fatigability typically involves an objective measurement of motor performance, such as decline in force or time-to-failure of a sustained task (Hunter, 2018). Perceived fatigability is often quantified via self-reported questionnaire items used to quantify its magnitude as well as its presence and can be assessed either at rest or during the exercise (Glynn et al., 2015; Enoka and Duchateau, 2016).

**FIGURE 1:**
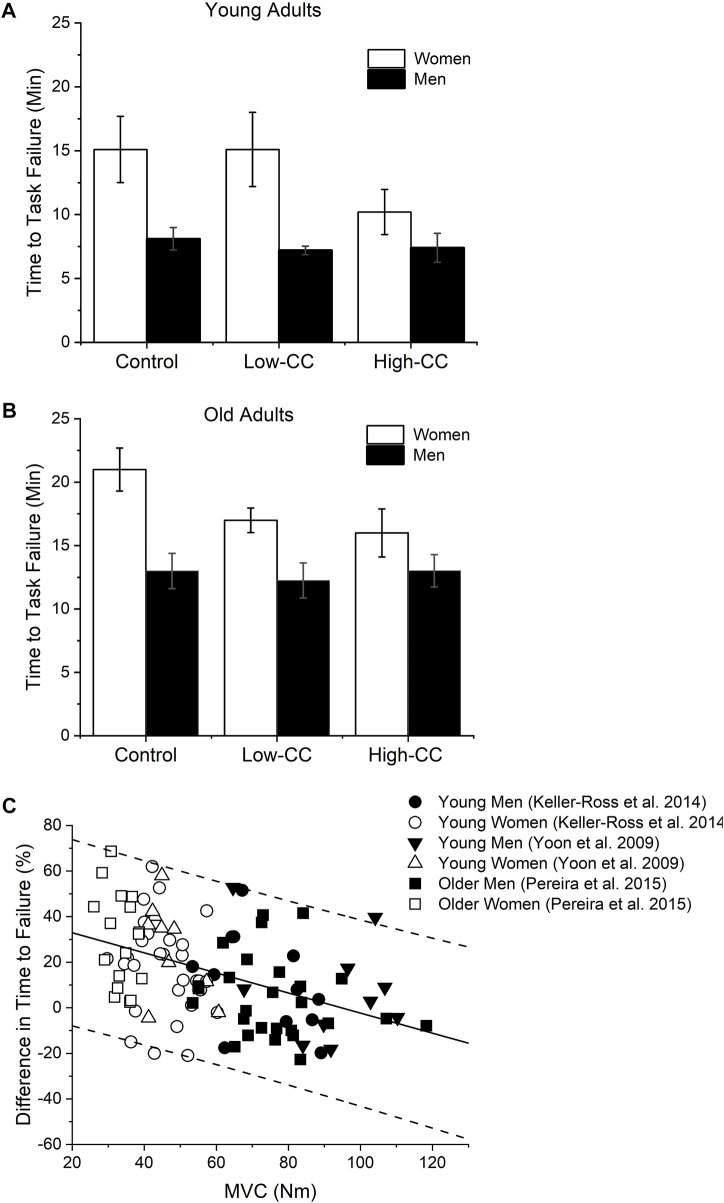
Time-to-task failure during control, low-cognitive challenge (low-CC, subtracting by 1) and a high-cognitive challenge (High-CC, subtracting by 13) during a low-to-moderate intensity isometric contraction (20% of maximal voluntary force (MVC)) with the elbow flexor muscles performed at different test sessions for young **(A)** and older **(B)** men and women. There was a reduction in time-to-task failure during the high-CC compared with control for young and older women, but not men (session × sex: *p* < 0.05 for both age groups). Low-CC reduced time-to-task failure for older women only (session × sex: *p* < 0.05). During control and low-CC sessions women had longer time-to-task failure than men for young and older groups (sex effect: *p* < 0.05). Data on C shows that initial maximal voluntary isometric contraction (MVC) torque of the elbow flexor muscles was negatively associated with the relative difference in time-to-task failure between the control and the high-CC session (all groups pooled: *r* = - 0.44, *p* < 0.001, n = 105). Older women are weaker than other groups and the magnitude of reduction in time-to-task failure with imposition of high-CC was larger in this group. In C the regression line is solid and the 95% predicted interval is indicated as dotted lines. In C values above zero in the *y*-axis indicate reduction in time-to-task failure in presence of the high-CC. Data from Keller-Ross et al. (2014) J Appl Physiol, Yoon et al. (2009) J Appl Physiol and Pereira et al. (2015) Clin Orthop Relat Res were used to construct the figures.

Some work-related tasks and activities of daily living are performed for prolonged periods in the presence of cognitive demand and stress. To understand the effects of cognitive demand and stress on motor fatigability, the time-to-task failure of sustained static and intermittent contractions was determined while individuals simultaneously solved a challenging mental math designed to increases the perceived stress (i.e., high-cognitive challenge) (Yoon et al., 2009; Mehta and Agnew, 2011) with distinct results depending on the limb used by young and older men and women. For example, for the elbow flexor muscles, young women, but not young men, had a brief time-to-task failure for a sustained fatiguing task (i.e., were more fatigable) for a high-cognitive challenge (subtractions by 13s) compared with a low-cognitive challenge (i.e., subtractions by 1) or control task (no math) (Figure 1A) (Yoon et al., 2009; Keller-Ross et al., 2014a). Conversely, for the lower extremity muscles (i.e., dorsiflexor muscles), no reduction in time-to-task failure in presence of either cognitive challenges (i.e., subtractions by 1 or 13) was observed in young men or women (Vanden Noven et al., 2014).

Although not fully understood, there are some insights on the potential physiological mechanisms that might explain the sex differences in young adults and limb dependency on the reduction in time-to-task failure in the presence of a high-cognitive challenge (i.e., subtractions by 13). The increased cardiovascular response in the presence of the high-cognitive challenge was not associated with the magnitude of the reduction in time-to-task failure, regardless of the limb assessed (Yoon et al., 2009; Mehta and Agnew, 2011; Keller-Ross et al., 2014b; Vanden Noven et al., 2014; Pereira et al., 2015a). However, maximal strength was a predictor of the reduction in time-to-task failure in the presence of high-cognitive challenge (Figure 1C). Notably, the ability of the central nervous system to activate the muscles was similar between the control and high-cognitive challenge conditions with no sex differences (Keller-Ross et al., 2014a). To further examine the influence of strength on the reduction in time-to-task failure in the presence of high-cognitive challenge, Keller-Ross et al. (2014) pooled the data from 75 participants and showed that weakest individuals, independent of sex, had larger reductions in time to task failure in presence of high cognitive challenge compared with stronger individuals. Additionally, the sex differences in time to task failure in presence of high-cognitive challenge was not present when maximal strength was entered as a covariate in the statistical analysis (Keller-Ross et al., 2014a). Thus, strength rather than sex-based differences, was responsible for the brief time-to-task failure in presence of high-cognitive challenge.

Limb differences in maximal strength may also explain the minimal reduction of time-to-task failure in presence of high-cognitive challenge (i.e., subtractions by 13) for the low extremity muscles but not upper extremity muscles. Specifically, the initial maximal force obtained on the upper extremity muscles (i.e., elbow flexors) was larger than lower extremity muscles (i.e., tibialis anterior) and the magnitude of sex differences in strength was also larger for the upper extremity compared with the lower extremity (Yoon et al., 2009; Vanden Noven et al., 2014). Combined, these findings suggest that mechanisms involved in the production of force during a fatiguing contraction, which are independent of sex or the ability of the central nervous system to activate the muscles, as partially responsible for the reduction in time-to-task failure in presence of a high-cognitive challenge. This is in accordance with previous observations in young adults showing that strength and its mechanisms were altered when a difficult cognitive task (i.e., incongruent Stroop color word task) was performed before a handgrip task (Bray et al., 2008; Bray et al., 2012).

Age-related decrements in maximal force (Hunter et al., 2016) and the role of initial strength in predicting increased fatigability with a cognitive challenge has consequences for healthy older individuals (i.e., >65 years old), particularly older women who are typically weaker than men (Charlier et al., 2016). Accordingly, for the upper extremity muscles, healthy older women had a larger magnitude of reduction in time-to-task failure in the presence of high-cognitive challenge (i.e., subtractions by 13) compared with healthy older men or young adults (Figure 1C). Older women were also vulnerable to increased fatigability (i.e., reduction in time-to-task failure) with the imposition of a low-cognitive challenge (simple mental math of subtracting by 1) with no reduction in time-to-task failure in the young men and women, and older men (Figure 1B) (Pereira et al., 2015a). For older individuals, sex differences in the presence of a high-cognitive challenge were also limb dependent, with a more brief time-to-task failure in the presence of a high-cognitive challenge for the upper extremity but not for the lower extremity in comparison with a control session (Vanden Noven et al., 2014; Pereira et al., 2015a). In older individuals, the magnitude of sex differences in maximal force for the upper extremity was larger compared with lower extremity, supporting the role of strength-related mechanisms on the reduction in time-to-task failure in presence of high-cognitive challenge.

In the lower limb experiments, however, a key component of the age-related decrements in motor performance was exposed. The high-cognitive challenge (i.e., subtractions by 13) increased the between-subject variability in performance fatigability of the lower extremity muscles for older adults compared with young individuals. This was seen as a much larger reductions of the time-to-task failure in the presence of a high-cognitive challenge for some older individuals but not others, with the group average not changing (Vanden Noven et al., 2014). The greater variability in motor performance with aging can often mask age-related differences when group average results are reported (Hunter et al., 2016). The imposition of a cognitive challenge during a motor task has the potential to expose these subtle individual differences in physiology and motor performance that are not always detected during control conditions.

## Cognitive challenge and force steadiness

In the laboratory setting, force steadiness during static tasks can be quantified with the fluctuations in force output normalized by the average of target force [Coefficient of variation (CV)] (Enoka and Farina, 2021). During control conditions (i.e., without the imposition of a cognitive challenge), force steadiness is reduced at very low compared to moderate levels of contraction, commonly referred as signal dependent noise (Harris and Wolpert, 1998; Stein et al., 2005). Older individuals and women are typically less steady (i.e., greater CV of force) than young adults and men respectively, for several different muscles of the upper and lower extremity muscles (Galganski et al., 1993; Tracy and Enoka, 2002; Tracy, 2007; Pereira et al., 2018), although there were no sex differences in steadiness during trunk flexion with the abdominal muscles (Deering et al., 2017). This is important because optimal performance in functional tests that resemble daily activities and work related tasks is largely determined by force steadiness and cognitive function (Ashendorf et al., 2009; Marmon et al., 2011; Mello et al., 2013; Müller et al., 2015; Davis et al., 2020). These daily and work activities often require increased cognitive flexibility, working memory and executive function (Kalechstein et al., 2003; Müller et al., 2015; Fisher et al., 2017). We and others have shown that force steadiness decreased when individuals characterized at a normal range of cognitive function simultaneously performed cognitive tasks targeting these cognitive constructs, such as math, n-back task, or auditory choice reaction tasks simultaneously with the motor task (Noteboom et al., 2001; Lorist et al., 2002; Voelcker-Rehage et al., 2006; Pereira et al., 2015b).

**FIGURE 2:**
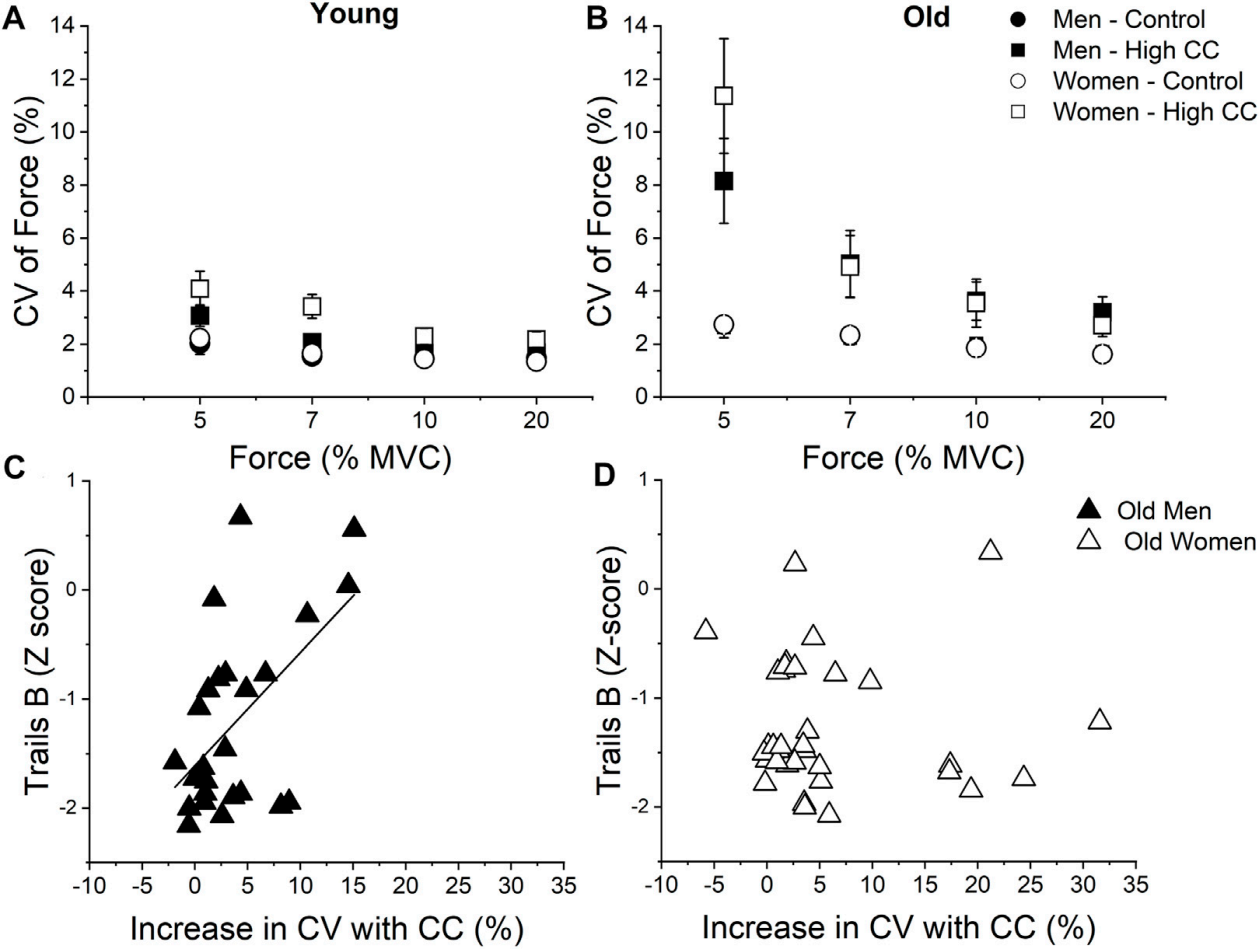
Coefficient of Variation (CV) of force for young **(A)** and older **(B)** individuals during control and high cognitive challenge (High-CC, subtracting by 13) trials at different force levels performed with the elbow flexor muscles. Large CV of force values indicate less steadiness. Older individuals had larger increases in CV of force in presence of high-CC compared with young individuals, particularly at very low forces (cognitive challenge × age group × force level: *p* < 0.05). Data is mean and standard error. **(C, D)** show the increase in CV of force of the elbow flexor muscles with imposition of high-cognitive challenge (CC) was associated with the results of trails B relative to normative values (i.e., Z-score) for older men but not older women (*r* = 0.55, *p* < 0.01 vs. *r* = 0.12, *p* = 0.50, respectively). Negative Z-score values indicate the performance is better than normative data. Data for A and B is from Hunter et al., 2016 J Appl Physiol and Pereira et al., 2015 Eur J Appl Physiol. **(C, D)** was constructed with data from Pereira et al., 2018 Front Physiol.

The magnitude of impairment in force steadiness in the presence of the cognitive challenge (i.e., subtraction by 13s) was larger at very low force levels, such as 5% of maximum than at moderate levels of contraction (i.e., 30–40% of maximum) (Pereira et al., 2015b). Age and sex differences were also observed and older individuals, particularly older women, exhibited larger impairments in force steadiness (larger fluctuations in force) than older men and young adults, respectively (Figures 2A, B). Older women were also more vulnerable than older men. This was observed when a low-cognitive challenge (i.e., subtraction by 1) reduced force steadiness in older women but not the young adults or older men (Yoon et al., 2009; Pereira et al., 2015b). Combined, these findings indicate that imposing a cognitive challenge (i.e., mental math) during the motor task can expose age-related reductions in force steadiness, particularly at very low force levels, with a greater magnitude in the reductions in women.

The mechanisms for the force fluctuations during a static contraction involve variability in the signals from the central nervous system to the skeletal muscle. Experiments without a cognitive challenge using recent advances in non-invasive technology, such as the ability to quantify motor unit behavior using high-definition electrodes (Farina et al., 2016), observed that force steadiness was largely influenced by the variability in the common synaptic input to motor units in young individuals (Negro et al., 2009; Farina and Negro, 2015). Using this technology, we observed that older individuals have greater variability in the common synaptic input to motor units during control conditions (i.e., no cognitive task), and the imposition of a high-cognitive challenge (i.e., subtractions by 13s) during very low forces (5% of maximum) increased the variability in the common synaptic input to motor units and more so in older adults than young. Additionally, the increased variability in common synaptic input induced by this high-cognitive challenge was associated with reductions in force steadiness (Pereira et al., 2019).

Factors influencing the increased variability of common synaptic input to motor units and impaired force steadiness, particularly at very low force levels, with the imposition of a high-cognitive challenge potentially involve disruptions in cortical mechanisms that are yet to be fully understood. The high-cognitive challenge can alter the oscillations in the effective neural drive to motor neuron pool, as observed by increased oscillatory activity between cortex and muscle (i.e., corticomuscular coherence) when a difficult mental math was imposed during very low intensity contractions of the hand muscles in older but not young individuals (Johnson and Shinohara, 2012). Further evidence of the cortical involvement was obtained from an experiment showing the magnitude of reduction in force steadiness of the elbow flexor muscles with the imposition of a high-cognitive challenge (i.e., subtractions by 13) was associated with poor performance on the Trail Making Test in older adults (Pereira et al., 2018) (Figures 2C, D). This test consists of sequentially connecting letters and numbers while maintaining accuracy as well as speed. It is typically used to provide insight into the individual executive function during neuropsychology assessments (Reitan, 1958; Strauss E., 2006). The predictive capacity of the Trail Making Test was maintained after accounting for normative results based on each individual age and education to minimize the potential effects of age-related reduction in executive function (Tombaugh, 2004). Because this study was conducted in healthy older adults, these observations highlight the fact that reductions in force steadiness in the presence of increased levels of cognitive demand are likely subclinical and can expose deficits in motor function with aging.

Additionally, the magnitude of influence of the Trail Making Test results on the impaired force steadiness with high-cognitive challenge was larger in older men compared with older women (Figures 2C, D), indicating potential sex differences in cortical mechanisms involved. Others have shown the Wechsler Abbreviated Scale of Intelligence, which provides the intelligence quotient, was associated with impairments in force steadiness of the knee extensor muscles during control conditions (i.e., without cognitive challenge) in individuals with multiple sclerosis (Gould et al., 2018). Considering the frequently described impairments in motor function when a cognitive task is performed simultaneously with a motor task in some clinical conditions, such as multiple sclerosis (Rooney et al., 2020), there is ample opportunity for high impact studies to identify the role of different domains of cognitive function on the impaired force steadiness in presence of heightened levels of cognitive demand.

The force steadiness obtained with laboratory-based tests using static contractions during control conditions is frequently associated with dynamic functional tasks of the upper and lower extremities that resemble activities of daily living (Kornatz et al., 2005; Marmon et al., 2011; Mello et al., 2013; Almuklass et al., 2016; Davis et al., 2020). Regardless, it is yet to be understood if these associations for dynamic functional tasks occur in the presence of a cognitive challenge and mechanisms involved. Although there are several reports indicating the cognitive cost during walking (Patel et al., 2014; Papegaaij et al., 2017; Koren et al., 2022), there are technical limitations to assess activation of motor units during dynamic tasks. It is not known, for example, if the increased variability in the common synaptic input to motor units in the presence of high-cognitive challenge is observed during such dynamic tasks, nor is it understood the relevance of the variability in the signals from the central nervous system to the muscle during fast dynamic activities (Enoka and Farina, 2021).

## Summary

Imposing a cognitive challenge during very low and moderate intensity static contractions can further increase the differences in motor performance between young and older men and women. A difficult cognitive challenge executed simultaneously with a motor task can amplify age-related decrements in motor function including brief time-to-task failure and reduction in force steadiness especially in women. Emerging evidence indicates that cortical mechanisms are involved with the loss of force steadiness with the imposition of cognitive challenge and include more variable common synaptic input to motor units. Peripheral mechanisms associated with strength, however, are predictive of the brief time-to-task failure during static contractions in presence of a cognitive challenge. These findings have potential implications for an aging workforce considering that accident proneness and injuries from work-related tasks are associated with the magnitude of cognitive stress.
